# Meniscal allograft arthroplasty for the treatment of trapeziometacarpal arthritis of the thumb

**DOI:** 10.1007/s11552-014-9737-4

**Published:** 2015-01-22

**Authors:** Paul S. Shapiro, Edward Diao, Lynn M. Givens

**Affiliations:** 1 Department of Orthopaedic Surgery, William Beaumont Hospital, 3535W. Thirteen Mile Road Suite #744, Royal Oak, MI 48073 USA; 2 Oakland University William Beaumont School of Medicine, 2200 N Squirrel Road, Rochester, MI 48309 USA; 3 Michigan Orthopaedic Institute, 26025 Lahser Road, Second Floor, Southfield, MI 48033 USA; 4 University of California, San Francisco, 505 Parnassus Avenue, San Francisco, CA 94143 USA; 5 California Pacific Medical Center, 45 Castro Street, San Francisco, CA 94114 USA; 6 San Francisco Surgery Center, San Francisco, CA USA; 7 Department of Surgery, William Beaumont Hospital, 3601W. Thirteen Mile Road, Royal Oak, MI 48073 USA

**Keywords:** Allograft, Arthritis, Arthroplasty, Meniscus, Thumb

## Abstract

**Background:**

Arthritis at the trapeziometacarpal joint of the thumb is common. Several surgical options exist showing favorable results. We report the outcomes after interposition of allograft knee meniscus for thumb trapeziometacarpal arthritis.

**Methods:**

Twenty-three patients (25 thumbs) had surgery for thumb trapeziometacarpal arthritis using knee meniscal allograft tissue. Eleven thumbs had a minimum follow-up of 24 months, 2 thumbs had a minimum of 12 months, and 12 thumbs had less than 6 months. Disabilities of arm, shoulder, and hand (DASH) questionnaire scores, pain levels, grip strength, pinch strength, range of motion, and radiographic measurements were performed.

**Results:**

Between the preoperative and 24-month follow-up measurements, patient pain levels were reduced. There was a significant improvement in DASH scores. Comparisons between preoperative and postoperative strength measurements showed increase in grip strength and key pinch strength. Trapeziometacarpal subsidence was 5.5 %, and subluxation index measurements decreased 3.9 %. There was no clinical or radiographic evidence of foreign body reaction and no other complications occurred.

**Conclusions:**

The results of meniscal allograft arthroplasty are comparable to other surgical techniques for trapeziometacarpal arthritis with respect to pain, outcomes, strength, oppositional motion, complications, surgical time, cost, and return to work. The results suggest that meniscal allograft arthroplasty is a viable option in the surgical management of stages II and III arthritis of the TM joint. Further follow-up and clinical studies are warranted.

## Introduction

Arthritis at the trapeziometacarpal (TM) joint of the thumb is a common problem encountered by hand surgeons. The prevalence of symptomatic TM joint arthritis is approximately 25 % of women and 8 % of men [50]. Patients present to the hand surgeon with the complaint of pain at the base of the thumb. It is typically a deep aching type that worsens with activity. TM arthritis of the thumb is classified according to the original description of Eaton and Glickel [14].

Nonoperative management includes oral non-steroidal anti-inflammatory drugs, corticosteroid injections, and splinting. When symptoms persist, surgical treatment is recommended.

A variety of surgical procedures have been reported for the treatment of stages II–III TM arthritis of the thumb (Table 1). As indicated in Table 1, some of these procedures are also indicated for stage IV disease which involves pantrapezial arthritis. Favorable results with surgery have been reported using simple excision [11, 20, 23, 27, 38, 54, 56], hematoma and distraction arthroplasty (HDA) [19, 24, 33], arthrodesis [3, 6, 37], autogenous interposition arthroplasty [17], suspensionplasty [12, 31, 51], and autogenous interposition arthroplasty with ligament reconstruction (LRTI) [5, 15, 22, 34, 52, 57, 59]. Each of these procedures has advantages and disadvantages and has shown good clinical results. Concern for kinematic alterations at LRTI donor sites from autogenous interposition grafting [39] led to proposed procedures using non-autogenous synthetic interposition materials. These included silicone interposition [43, 49], orthosphere interposition (Wright Medical Technology, Inc.) [1, 4], and Artelon interposition (Artimplant USA) [41, 42]. However, some of these techniques were fraught with complications including implant instability, implant fracture, particulate synovitis, implant subsidence, and foreign body reactions [1, 4, 7, 21, 43, 44]. Because of some of the limitations of these techniques, allograft tissue procedures including acellular dermal matrix interposition and costochondral interpositional arthroplasty have been explored [32, 53]. The authors of this study propose the use of meniscal allograft tissue as a spacer and stabilizer of the arthritic TM joint. Reports of meniscal allograft transplantation in the knee have been favorable at 10-years follow-up [26]. Meniscus has also been reported in the treatment of glenohumeral osteoarthritis [10, 35, 58]. Nanavati et al. [40] reported on meniscal allograft insertion with proximal row carpectomy in a cadaveric study.

**Table 1:** Surgical treatment options for stages II–III TM arthritis of the thumb

I	Excisonal arthroplasty^a^
II	Hematoma distraction arthroplasty^a^
III	Arthrodesis
IV	Autogenous interposition arthroplasty^a^
V	Suspensionplasty^a^
VI	Autogenous interposition arthroplasty with ligament reconstruction (LRTI)^a^
VII	Joint replacement arthroplasty
VIII	Non-autogenous interposition arthroplasty
a.	Silicone interposition^a^
b.	Orthosphere interposition
c.	Artelon interposition
d.	Acellular dermal matrix allograft arthroplasty
e.	Costochondral allograft arthroplasty

^a^Also indicated for stage IV disease

The hypothesis for this study was that the use of knee meniscal allograft tissue is a viable option for the surgical treatment of TM arthritis of the thumb.

## Materials and Methods

The procedure design for meniscal allograft arthroplasty (MAA) was tested and refined using a cadaver model in the laboratory. Human Investigation Committee (HIC) approval was obtained from our Institutional Review Board.

Between 2009 and 2012, 25 consecutive MAA procedures were performed by the same surgeon (PSS) on 23 patients with stage III basal joint arthritis using an allograft knee meniscus transplanted into the thumb TM joint (Table 2). No adjunctive procedures were performed in the series at the time of surgery. There were 13 females and 12 males with a mean age of 57.5 years (range 42–77 years). Eighteen of the patients were employed, and seven were either retired or disabled. Twenty-four thumbs were diagnosed with osteoarthritis and one with traumatic arthritis. One patient could not be located to complete questionnaires and follow-up examinations. Eleven thumbs had a minimum follow-up of 24 months, 2 thumbs had a minimum of 12 months, and 12 thumbs had less than 6 months.

**Table 2:** Patient demographics

Patient no.	Age	Gender	Operated hand	Occupation	Diagnosis	Latest follow-up (months)
1	67	F	L	Retired	OA	27
2	77	F	L	Retired	OA	31
3	42	M	L	Auto worker	OA	31
4	43	F	R	Hair style	OA	32
5	42	M	R	Auto worker	OA	29
6	54	F	R	Nurse anesthetist	OA	30
7	62	M	R	Engineer	OA	24
8	68	M	L	Salesman	OA	25
9	63	M	L	Engineer	OA	12
10	61	F	R	Art teacher	OA	26
11	48	M	L	Service consultant	Traumatic arth	8
12	58	M	R	Retired	OA	24
13	62	M	L	Security guard	OA	23
14	50	F	R	Office worker	OA	12
15	63	F	R	Cashier	OA	6
16	69	F	R	Office worker	OA	6
17	48	F	L	Disabled	OA	1
18	53	M	L	Salesman	OA	6
19	73	M	R	Retired	OA	7
20	53	F	R	Pharmacy technician	OA	8
21	59	F	R	Retired	OA	6
22	62	M	R	Retired	OA	6
23	55	F	L	Surgical nurse	OA	6
24	46	F	L	Legal assistant	v	3
25	57	M	L	Electrician	OA	3

### Clinical Evaluation

All patients presented with the complaint of pain at the base of the thumb as well as impaired function. Physical examination revealed tenderness to palpation at the TM joint and pain with axial loading of the thumb. Posteroanterior radiographs of the hand demonstrated joint space narrowing at the TM joint without evidence of narrowing at the scaphotrapezial joint (ST) (stage III). All patients were initially treated nonoperatively for a minimum of 6 months with splinting, oral anti-inflammatory medications, and with intra-articular steroid injections. Activity modification was encouraged.

### Preoperative Data Collection

The disabilities of arm, shoulder, and hand (DASH) questionnaire was utilized as a preoperative outcomes measure of the patient’s symptoms and functional status [2, 28].

Pain was evaluated using a visual analog scale (VAS) with 0 representing no pain and 10 representing the highest degree of pain. The visual analog scale measurements were recorded at maximal loading in key pinch as reported by Nilsson et al. [41]. The original pain scale (from 1 to 10, with 0 being no pain and 10 being the most severe pain) was transformed to a 4-point scale with 0 = no pain, 1–3 mild pain, 4–7 moderate pain, and 8–10 severe pain.

Additional preoperative data for all patients included grip strength using a Jamar dynamometer (Asimov Engineering, Los Angeles, CA) and tip, key, and palmar pinch strength of the involved hand using a pinch meter (Therapeutic Instruments, Clifton, NJ). Preoperative range of motion data for all patients included carpometacarpal (CMC), metacarpophalangeal (MP), interphalangeal (IP), and oppositional (OPP) range of motion. OPP was measured by assessing the thumb tip ability to touch an anatomic landmark at the base of the small finger.

### Operative Procedure

**Fig. 1:**
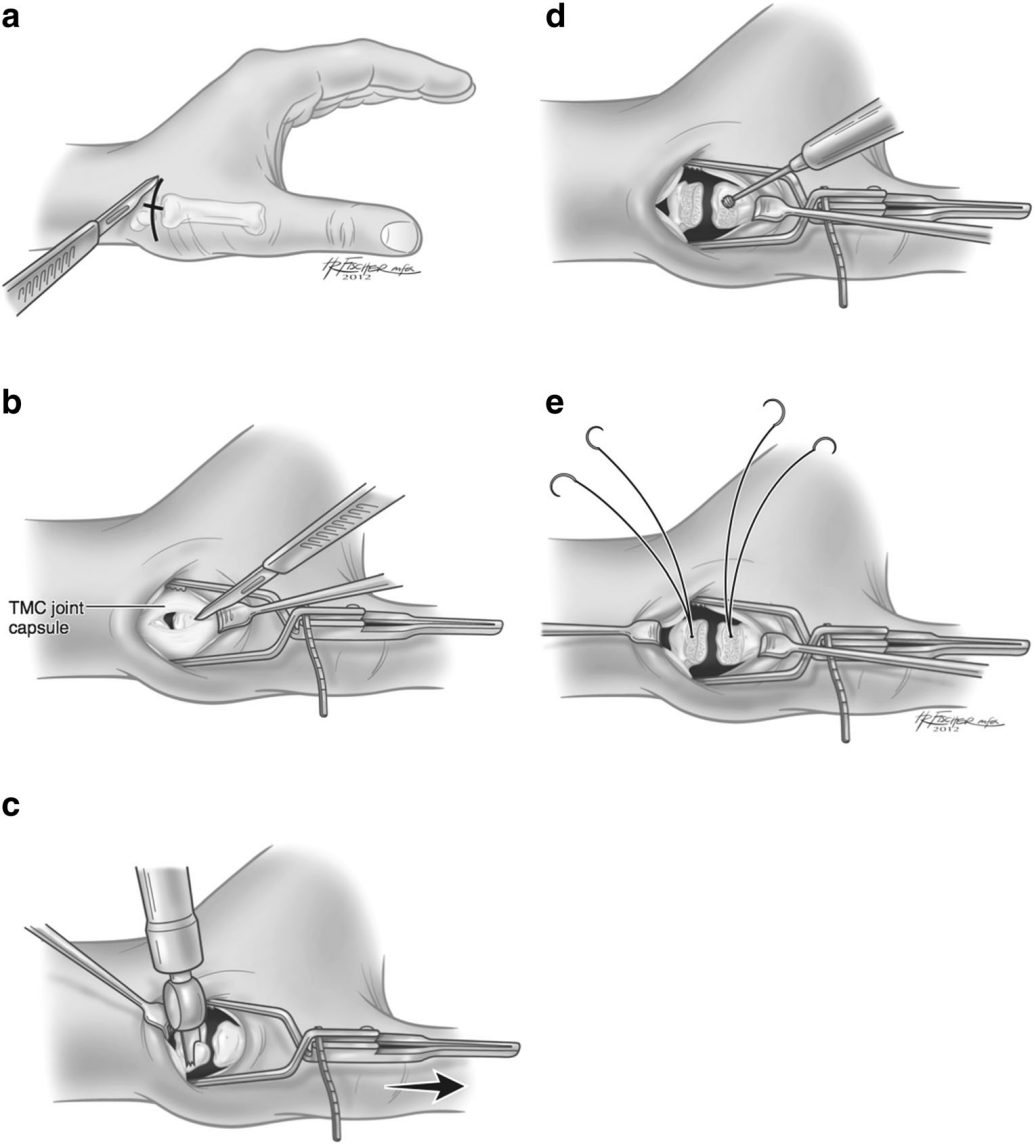
**a**–**e** Operative procedure 1

Anesthesia consisted of a regional block induced prior to surgery along with sedation. A long-acting pain pump was used for all patients in the series, but is no longer currently used as it is felt not to be necessary. A dorsoradial incision was made obliquely over the TM joint (Fig. 1a). The superficial branches of the radial nerve were identified and protected. An interval between the abductor pollicis longus (APL) and extensor pollicis brevis tendons was dissected. The radial artery was identified proximally and protected by mobilizing it dorsally. A longitudinal incision was made through the TM joint capsule ulnar to the APL insertion extending 1.5 cm proximal and distal to the joint to create two periosteal sleeves (Fig. 1b). A small oscillating saw was used to remove 2–3 mm of distal trapezium (Fig. 1c). The dorsal cortex of both the trapezium and the proximal metacarpal were then removed using a small burr or a rongeur (Fig. 1d). Absorbable suture anchors holding 2–0 Fiberwire (Arthrex, Inc., Naples, FL) with two tapered needles at the suture ends were inserted into the trapezium and the metacarpal base (Fig. 1e).

**Fig. 2:**
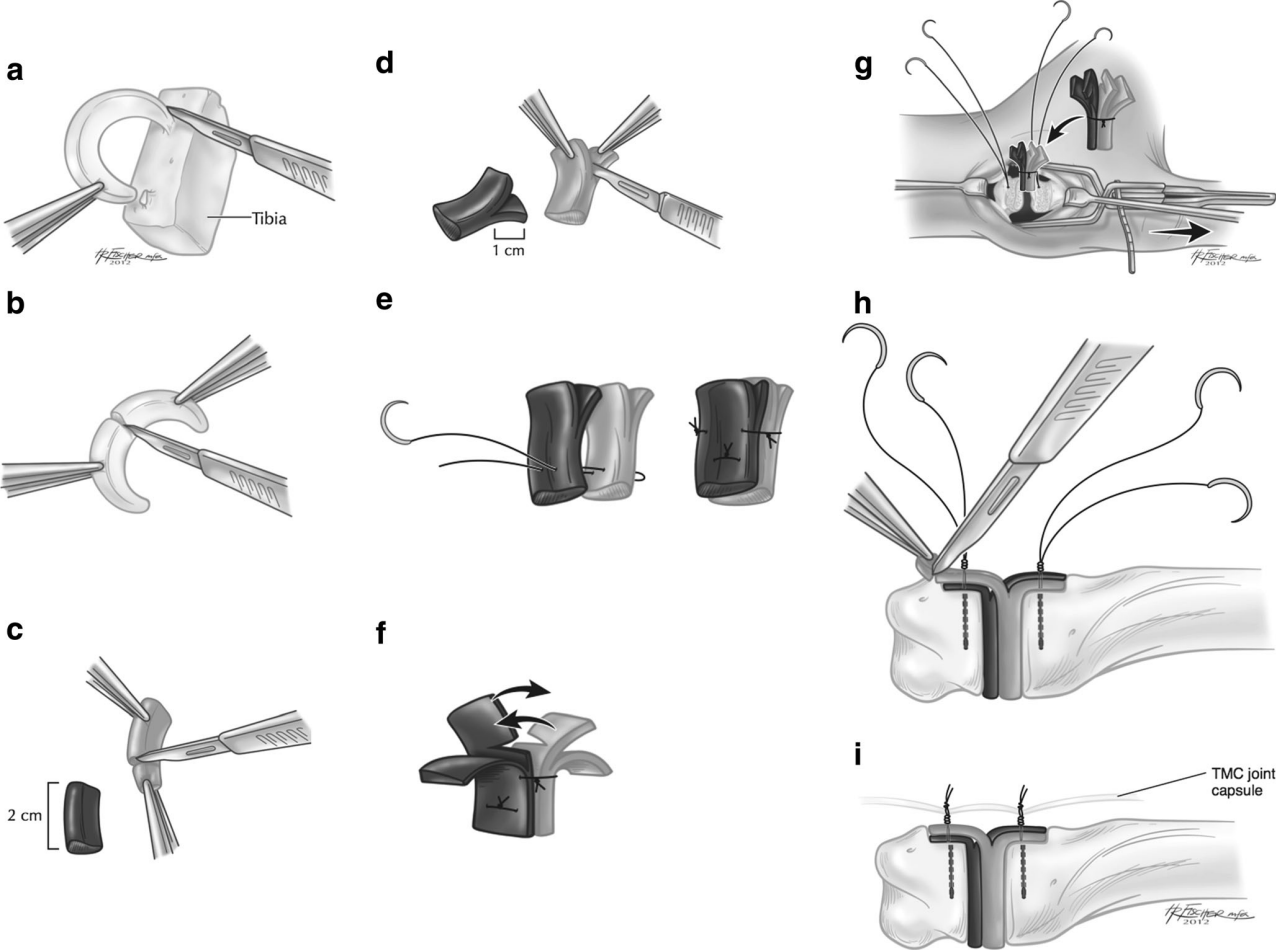
**a**–**i**: Preparation of meniscus

Attention was then shifted to preparation of the meniscal allograft for insertion in to the thumb TM space. The graft tissue was placed in a normal saline bath for 5 min to allow it to thaw. A minimum meniscal graft height of 5 mm was a prerequisite for use. The meniscal horns were removed from the tibia fragment (when present) (Fig. 2a). The allograft was then cut transversely into two equal halves (Fig. 2b). Each fragment was then cut to form two rectangular shaped parts measuring 2 cm in length (Fig. 2c). Each part was then cut in the coronal plane for half of its length to create two 1-cm long flaps or “wings” (Fig. 2d). Three fiberwire sutures were then used to connect the two parts leaving the wider portions of the triangular meniscus facing outward creating a rectangular shape to the graft now in two planes (Fig. 2e). Two small incisions were then made at the base of the two inner flaps to allow them to lay flat as the top of the “T” was created (Fig. 2f).

The base of the graft was then inserted into the TM joint (Fig. 2g). The suture anchor FiberWires were then passed through the wing fragments of both sides of the graft and tied secure. Graft tissue outside of the previously created trapezium and metacarpal footprints was then removed (Fig. 2h). The suture anchor knots were not cut, but rather were used to secure the capsular flaps over the meniscal graft (Fig. 2i). The joint was then compressed and taken through a full range of passive motion to insure smooth tendon gliding, and adequate joint stability. Flouroscopic radiographs were obtained to ensure adequate positioning of the joint and implant. After wound closure, a bulky thumb spica bandage and short-arm fiberglass splint were applied. All patients were discharged from the hospital or surgery center on the day of surgery and were prescribed oral pain medications to be used as needed.

Postoperative management included thumb Spica casting applied 1 week postoperative and continued for 6 weeks, followed by 6 weeks of thermoplastic splinting and occupational therapy. The 6-week immobilization period was instituted for comparison with other procedures described in the literature. The authors currently use a 3-week period of casting followed by 3–6 weeks of splinting. Occupational therapy is no longer routinely prescribed, and is currently used only when deemed necessary.

### Postoperative Data Collection

Data was collected preoperatively and at 3 months, 6 months, 1 year and 2 years postoperative. Recorded data included DASH scores, pain level, grip strength, tip, key and pinch strength, CMC, MP, IP, and OPP range of motion.

Radiographic analysis of the space between the metacarpal base and proximal trapezium without stress were calculated preoperatively, immediately postoperative (less than 2 weeks), and at latest follow-up. Measurements of the thumb TM joint space were indirectly measured by calculating the trapeziometacarpal index (TMI) as previously described [29, 46, 53].

Subluxation the thumb TM joint (S) along with the subluxation index (SI) was measured as described by Trumble et al. [53].

### Data Analysis

A Gamma statistic was used to assess the strength of the association between pain and time. A 95 % confidence interval for this statistic was calculated. *P* values and 95 % confidence intervals were calculated for the differences between pre- and post-surgical values with *p* values less than an alpha of 0.05 considered statistically significant. The statistical analysis used SAS 9.2 for Windows system and R software for the pain graph.

## Results

### Pain

**Fig. 3:**
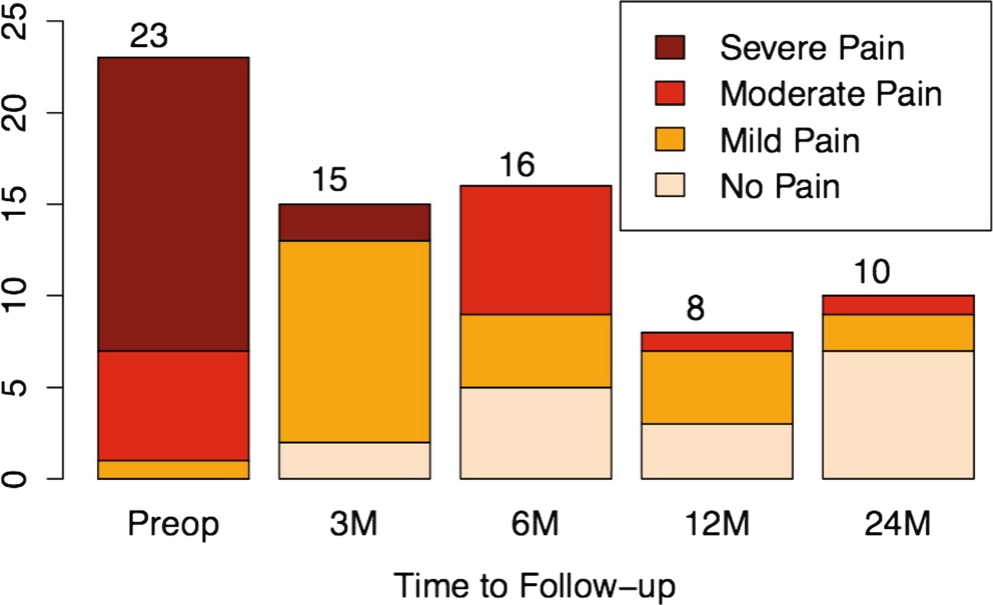
Pain distribution at follow-up. The gradual shift, from severe and moderate pain to no pain, can be seen as the colors change intensity. The *numbers at the top of the bars* represent the total number of subjects at that time point (preop = before surgery, 3M = 3 months, 6M = 6 months, 12M = 12 months, 24M = 24-months follow-up)

Preoperative pain levels on these patients averaged 7.7 and were significantly reduced to 0.9 at 24-months follow-up: this change was statistically significant (*p* = 0.002). Preoperatively, pain scores of severe and moderate levels predominated (96 % out of 23 patients), and then gradually changed over 24-months follow-up where 91 % had no pain or mild pain (Fig. 3). At 6-months follow-up 59 % had no pain or mild pain.

### Outcomes Data

**Fig. 4:**
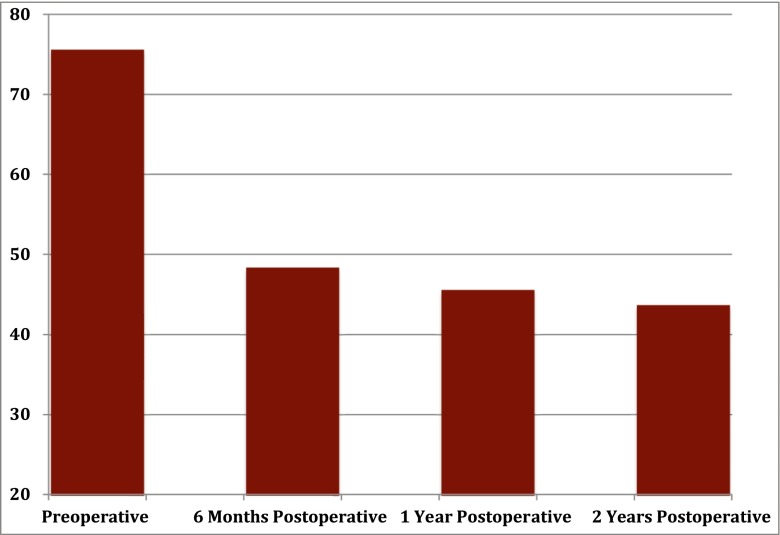
Dash scores as measured at four time points

There was a significant improvement in the DASH scores of these patients from a mean of 75.6 preoperatively to 43.7 (42.1 % improvement) at 24-months follow-up (Fig. 4).

### Strength and Motion

Improvements were seen in grip strength (28.6 %), tip (57.3 %), key (31.4 %), and palmar (33.8 %) pinch strength. These were not statistically significant (Table 3).

**Table 3:** Qualitative and quantitative statistical data

Feature	*n*	Preoperative	*n*	Postoperative	%Change	95 %	*p* value	*n*
Dash score	23	75.6 ± 21.0	10	43.7 ± 19.4	(+) 42.1	[−43.7, −12.8]	0.0026	11
Grip strength (kg)	21	24.4 ± 15.4	10	31.4 ± 15.9	(+) 28.6	[−7.3, 8.8]	0.8	11
Tip pinch (kg)	21	3.4 ± 4.5	10	5.3 ± 2.4	(+) 57.3	[−0.5, 2.9]	0.14	11
Key pinch (kg)	21	4.9 ± 2.3	10	6.4 ± 2.4	(+) 31.4	[−1.0, 1.8]	0.5	11
Palmar pinch (kg)	21	3.8 ± 2.3	10	5.7 ± 2.1	(+) 33.8	[−1.2, 3.5]	0.3	11
Abduction	21	48.8 ± 8.9	10	45 ± 0				11
MCP joint ROM	21	46.2 ± 15.8	10	38.0 ± 10.6	(−) 17.7	[−27.8, −5.4]	0.008	11
IP joint ROM	21	56.5 ± 17.1	10	77.0 ± 10.9	(+) 26.6	[7.1, 30.8]	0.005	11
Trapeziometacapal index (TMI)	21	57.9 ± 12.5	20	54.7 ± 11.4	(−) 5.5	[−3.4, −0.3]	0.02	19
Subluxation index (SI)	21	5.1 ± 2.0	20	4.9 ± 2.7	(−) 3.9	[−1.3, 0.83]	0.7	19

Mean ± SD are provided for pre-operation and 24-months follow-up for DASH scores, grip strength, tip, key, palmar pinch strength, thumb abduction, MCP and IP range of motion. The after surgery data for TM distance and subluxation refers to values obtained from radiographs taken at the most recent follow-up. The 95 % confidence intervals and *p* value correspond to the *t* test of the difference (after-before surgery) in scores

A significant improvement was seen in IP range of motion of 26.6 %, and a significant reduction was seen in MCP motion of 17.7 %. Thumb abduction decreased (7.7 %) but was not statistically significant. All patients were able to touch their thumb tip to the base of the small finger MCP joint at 6-month follow-up (Table 3).

### Radiographic Data

The mean TMI values were found to significantly decrease from preoperative to latest follow-up by a mean of 3.2 mm (5.5 %). Mean SI values decreased by 0.2 mm (3.9 %), but were not statistically significant (Table 3).

There was no evidence of trapezial or metacarpal bone osteolysis or cyst formation on the latest follow-up radiographs.

### Complications

There were no other complications noted including infection, RSD, or postoperative paresthesias. There were no cases of post-operative pain syndrome.

### Return to Work

All 18 of the employed patients at the time of surgery returned to their previous occupations without restriction within 3–6 months postoperative.

### Surgical Time

The mean surgical time from incision to splint application was 78 min.

### Cost

**Fig. 5:**
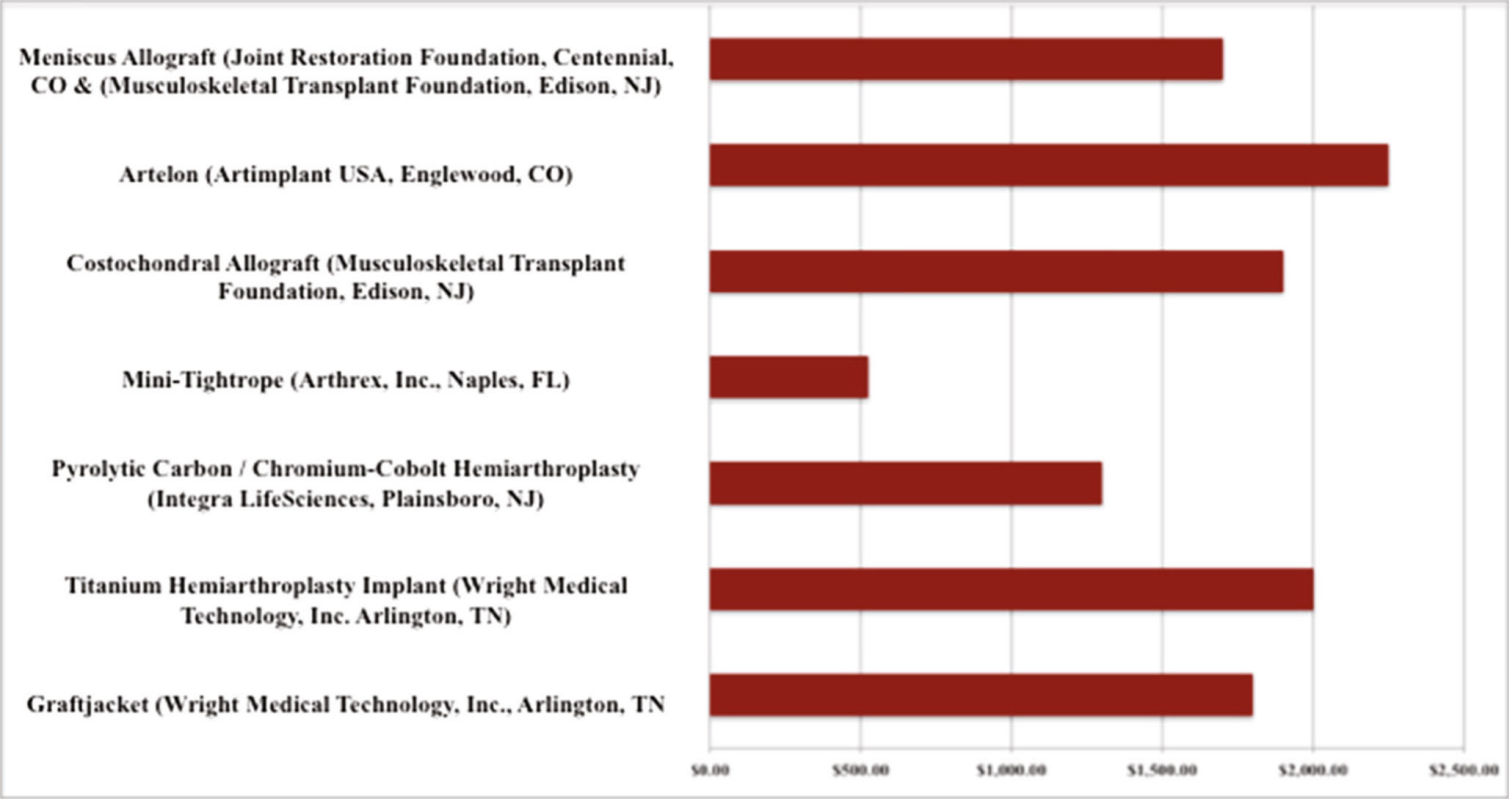
Meniscal allograft tissue costs compared to other commercially available implants

The meniscal allograft tissue cost was comparable to other commercially available implants (Fig. 5).

## Discussion

The operative procedure for MAA was designed to be similar to that of the Artelon procedure [41]. In contrast with studies demonstrating foreign-body reactions to the Artelon spacer [7, 21, 44], short-term follow-up with the MAA procedure did not show evidence of this complication. As with Artelon, MAA has the intention of achieving TM joint capsule augmentation (by the horizontal portion of the allograft), and resurfacing of the TM articular surface (vertical portion of the allograft). The placement of the meniscal allograft “wings” over the dorsal aspect of the TM joint may augment the dorsal ligament complex which has been found to play a significant role in TM joint stability [16].

### Pain

The results for pain using MAA compare with studies previously reported for TM arthroplasty using other techniques. Our results of 91 % with no pain or mild pain at final follow-up are similar to previous reports including 89–95 % satisfaction in patients undergoing LRTI [5, 34, 52], 91 % in patients undergoing HDA [24], 94 % for suspensionplasty [31], and 95 % for costochondral allografting [53].

### Outcomes Analysis

The significant improvement of DASH scores in this study compares with other studies using this and other outcomes measures with similar improvements noted [2, 8, 24, 28, 31, 33, 46, 53].

### Strength and Motion

Our results showing improved strength variables compare well with other techniques. Similar improvements in grip and pinch strength have been reported in patients undergoing LRTI, HDA, arthrodesis, suspensionplasty, and Artelon procedures [25, 31, 33, 34, 41, 46, 52, 55, 59].

In our study, 21 out of 21 patients available at 6-months follow-up were able to oppose to the base of the small finger which compares well to other techniques. Similarly, Kuhns et al. [33] reported 96 % of patients undergoing HDA could oppose to the base of the small finger by 6 months after surgery. In contrast, Tomaino et al. [52] reported a 2-year follow-up on patients who had LRTI and noted 7 of 25 (28 %) could not touch the base of the small finger. Similarly, Yang and Weiland [59] reported on 15 patients after LRTI and noted that 33 % could not oppose to the base of the small finger at 32-months follow-up.

### Radiographic Analysis

**Fig. 6:**
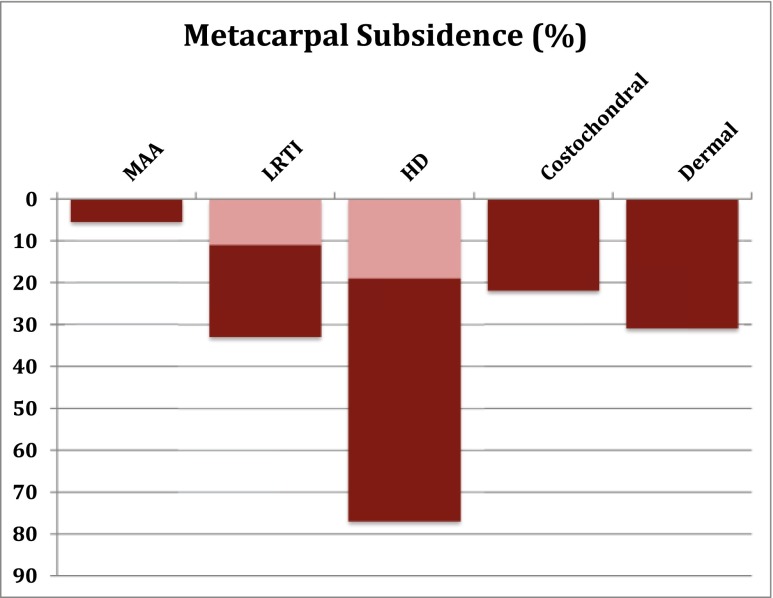
Metacarpal subsidence at latest follow-up as reported in the literature. *Double shading* represents variable reports. *MAA* = meniscal allograft arthroplasty, *LRTI* = ligament reconstruction and tendon interposition arthroplasty, *HDA* = hematoma distraction arthroplasty, *Costochondral* = costochondral allograft arthroplasty, *Dermal* = accellular dermal interposition arthroplasty

The significance of proximal migration of the thumb metacarpal after TM arthritis surgery with regard to overall clinical outcomes remains controversial. Reports on the techniques of LRTI, suspensionplasty, HDA, acellular dermal allograft, and costochondral allograft have shown evidence of proximal migration at rest and with stress varying from 11 to 77 % [5, 24, 32–34, 46, 47, 52, 53, 59]. Radiographic analysis was not performed by Nilsson et al. [41] preventing direct comparison to the Artelon in this respect. The MAA procedure showed a 5.5 % proximal migration based on non-stress x-ray analysis (Fig. 6). As is the case for costochondral arthroplasty, the finding of less subsidence with MAA is due to the need for only a partial trapeziectomy in comparison with LRTI and HDA where a complete trapeziectomy is performed. To date, no reports have shown a negative correlation between strength or functional outcome in association with metacarpal subsidence using either static or stress radiographs [18, 19, 24, 33, 46, 52]. With 6-years follow-up after either simple trapeziectomy or trapeziectomy + LRTI, Salem and Davis [45] reported evidence of scaphoid-metacarpal degenerative changes in 28 and 3.4 %, respectively. However, the presence of degenerative changes did not adversely affect clinical outcome.

Reports of metacarpal subluxation relative to the trapezium are limited. Tomaino et al. [52] reported an 8 % subluxation rate on stress radiographs at 6 years following LRTI. Trumble et al. [53] reported a 5 % reduction in subluxation following costochondral allograft, but did not comment on its significance. Although not significant, this study showed a decrease in subluxation of 3.9 % based on the SI. Dorsal placement and capsular closure over the meniscal graft in MAA may prevent metacarpal subluxation.

### Complications

There were no complications noted in this study and only one patient (4 %) required revision surgery after a significant postoperative injury. These findings are comparable to previous studies. LRTI and HDA have reported up to 9 % complication rates including temporary paresthesias in the superficial branch of the radial nerve, superficial pin tract infection, and deep infection [24, 33, 34]. Complications requiring additional surgery have been reported at 0 % for arthrodesis [25], 0–3.3 % for LRTI [5, 34, 59], 0 % for HDA [33], 0 % for suspensionplasty [31, 47], 5.5–10 % for Artelon interposition [41, 42], 2.4 % for acellular dermal allograft arthroplasty [32], and 4.3 % for costochondral allograft arthroplasty [53].

Reports using synthetic materials including silicone, polyurethane, polytetrafluoroethylene (Gore-Tex), and polypropylene (Marlex) have been associated with foreign body reactions and have largely been abandoned [9, 30, 36, 48]. Similarly, foreign body reactions have been reported after use of the Artelon implant [7, 21, 44], although some of these reports have had commentary with disclaimers [13]. In contrast, foreign body reactions have not been reported after costochondral allograft implantation [53], or after acellular dermal allograft implantation [32]. Foreign body reactions have also not been reported in procedures done in the knee and shoulder using meniscal allograft [26, 35, 58]. It would appear that the use of human allograft tissue in the treatment of TM arthritis may have the advantage of not causing adjacent foreign body reactions as seen with synthetic implants.

### Surgical Time

Surgical time for MAA averaged 78 min. This compares favorably to average surgical times reported by Sandvall et al. [46] for LRTI and HDA of 125 and 71 min, respectively. The MAA technique is similar to that of the Artelon procedure in terms of time, with preparation of the meniscus adding 10–15 min to the overall procedure.

### Return to Work

The results of this study suggest that MAA may be indicated for high- or low-demand patients, as all employed patients, including an auto worker and a nurse anesthetist returned to their previous occupations within 6 months of surgery. Long-term studies comparing MAA to LRTI and/or HDA in an active male population may be useful to further address the impact of metacarpal subsidence or lack thereof in this subpopulation.

With the acknowledged limitations of this study having low patient numbers and short-term follow-up, the results of MAA are comparable to other surgical techniques for TM arthritis with respect to pain, outcomes, strength, oppositional motion, complications, surgical time, cost, and return to work. These results support the use of this technique in the surgical management of stages II and III arthritis of the TM joint. Further follow-up clinical studies are warranted. Refinement and simplification of the surgical technique and shortening of the time of immobilization to accelerate the rehabilitation schedule are areas we intend to explore in the future.
